# Bis[*N*′-(2-pyridylmethyl­ene-κ*N*)benzo­hydrazide-κ*N*′]bis­(thio­cyanato-κ*N*)cobalt(II)

**DOI:** 10.1107/S1600536809029006

**Published:** 2009-07-25

**Authors:** Amitabha Datta, Nien-Tsu Chuang, Jui-Hsien Huang, Hon Man Lee

**Affiliations:** a National Changhua University of Education, Department of Chemistry, Changhua, Taiwan 50058

## Abstract

In the title complex, [Co(NCS)_2_(C_13_H_11_N_3_O)_2_], the Co^II^ centre adopts a distorted octa­hedral coordination geometry with two *cis*-bidentate Schiff base ligands and two *cis* thio­cyanate ligands. The Schiff base ligand coordinates *via* the imine N and pyridine N atoms. The Co^II^ atom lies on a crystallographic twofold rotational axis. Non-classical inter­molecular C—H⋯O hydrogen bonds link the complex mol­ecules into chains along [001].

## Related literature

For metal complexes of the same Schiff base, see: Basak *et al.* (2008 ▶); Chen *et al.* (2005 ▶); Christidis *et al.* (1999 ▶); Pal & Pal (2002 ▶); Paschalidis & Gdaniec (2004 ▶); Paschalidis *et al.* (2000 ▶); Pelagatti *et al.* (2000 ▶); Pouralimardan *et al.* (2007 ▶); Ogata *et al.* (2008 ▶).
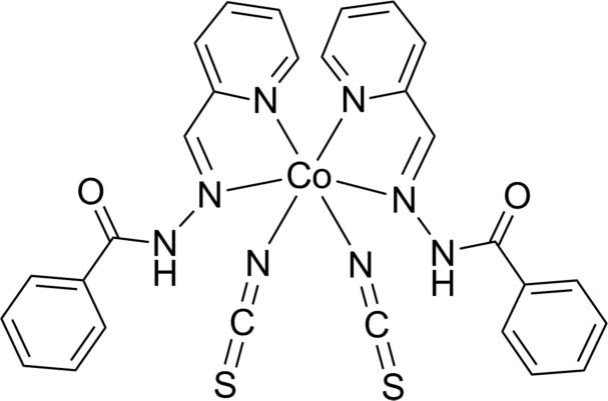


## Experimental

### 

#### Crystal data

[Co(NCS)_2_(C_13_H_11_N_3_O)_2_]
*M* *_r_* = 625.59
Monoclinic, 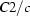

*a* = 17.112 (10) Å
*b* = 10.256 (6) Å
*c* = 17.945 (15) Å
β = 117.50 (3)°
*V* = 2794 (3) Å^3^
*Z* = 4
Mo *K*α radiation
μ = 0.81 mm^−1^
*T* = 150 K
0.19 × 0.18 × 0.10 mm

#### Data collection

Bruker SMART APEXII diffractometer
Absorption correction: multi-scan (*SADABS*; Sheldrick, 1996 ▶) *T*_min_ = 0.862, *T*_max_ = 0.924
15579 measured reflections
2742 independent reflections
1666 reflections with *I* > 2σ
*R*_int_ = 0.119

#### Refinement

*R*[*F* ^2^ > 2σ(*F* ^2^)] = 0.040
*wR*(*F* ^2^) = 0.100
*S* = 0.97
2742 reflections
186 parameters
H-atom parameters constrained
Δρ_max_ = 0.60 e Å^−3^
Δρ_min_ = −0.55 e Å^−3^

### 

Data collection: *APEX2* (Bruker, 2007 ▶); cell refinement: *SAINT* (Bruker, 2007 ▶); data reduction: *SAINT*; program(s) used to solve structure: *SHELXS97* (Sheldrick, 2008 ▶); program(s) used to refine structure: *SHELXL97* (Sheldrick, 2008 ▶); molecular graphics: *SHELXTL* (Sheldrick, 2008 ▶); software used to prepare material for publication: *DIAMOND* (Brandenburg, 1999 ▶).

## Supplementary Material

Crystal structure: contains datablocks I, global. DOI: 10.1107/S1600536809029006/pv2186sup1.cif

Structure factors: contains datablocks I. DOI: 10.1107/S1600536809029006/pv2186Isup2.hkl

Additional supplementary materials:  crystallographic information; 3D view; checkCIF report

## Figures and Tables

**Table 1:** Hydrogen-bond geometry (Å, °)

*D*—H⋯*A*	*D*—H	H⋯*A*	*D*⋯*A*	*D*—H⋯*A*
C13—H13⋯O1^i^	0.95	2.50	3.335 (5)	146

Symmetry code: (i) 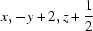.

**Fig. 1.**
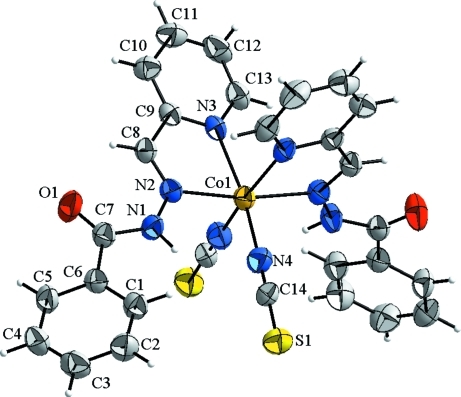
The structure of the title complex, showing 50% displacement ellipsoids for non-H atoms. The H atoms are dipicted by circles of an arbitrary radius. Unlabeled atoms of the complex are related to the labeled atoms by: -x, y, 0.5 - z.

**Fig. 2.**
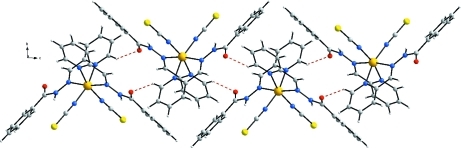
A packing diagram of the title compound along the a axis showing the intermolecular hydrogen bonds (dashed lines).

## supplementary crystallographic information

### Comment

The Schiff base, *N*'-(pyridin-2-ylmethylene)benzohydrazide, reacts with cobalt acetate tetrahydrate and sodium thiocyanate in water/methanol mixture to afford the tilte complex, (I). The cobalt atom lies on a crystallographic 2-fold rotational axis. The complex (I) adopts octahedral coordination geometry with the two bidentate Schiff base ligands being *cis* to each other (Fig. 1). The Schiff base coordinates *via* the imine N and pyridine N atoms. The two thiocyanate ligands are also *cis* to each other.

Metal complexes of the same Schiff base ligand have been reported in the literature (Basak *et al.* 2008; Chen *et al.* 2005; Christidis, *et al.* 1999; Pal & Pal, 2002; Paschalidis & Gdaniec, 2004; Paschalidis *et al.* 2000; Pelagatti *et al.* 2000; Pouralimardan, *et al.* 2007; Ogata *et al.* 2008).

Non-classical intermolecular H-bonds of the type C—H···O exist (Table 1). These H-bonds link the complex into one-dimensional hydrogen bonded chains (Fig. 2).

### Experimental

To a methanolic solution (20 ml) of cobalt acetate tetrahydrate (0.249 g, 1.00 mmol), a solution of *N*'-(pyridin-2-ylmethylene)benzohydrazide (1.00 mmol) was added, followed by the addition with constant stirring of a solution of sodium thiocyanate (0.162 g, 2.00 mmol) in a minimum volume of water/methanol mixture. The resultant solution was kept at room temperature yielding orange crystals suitable for X-ray diffraction after a few days. Crystals of (I) were isolated by filtration and were air-dried.

### Refinement

All the H atoms were positioned geometrically and refined as riding atoms, with C—H = 0.95 and N—H = 0.88 Å while *U*_iso_(H) = 1.2*U*_eq_(C or N) for all the H atoms.

### Crystal data

**Table:**

[Co(NCS)_2_(C_13_H_11_N_3_O)_2_]	*F*(000) = 1284
*M**_r_* = 625.59	*D*_x_ = 1.487 Mg m^−^^3^
Monoclinic, *C*2/*c*	Mo *K*α radiation, λ = 0.71073 Å
Hall symbol: -C 2yc	Cell parameters from 2526 reflections
*a* = 17.112 (10) Å	θ = 2.4–22.3°
*b* = 10.256 (6) Å	µ = 0.81 mm^−^^1^
*c* = 17.945 (15) Å	*T* = 150 K
β = 117.50 (3)°	Tubular, orange
*V* = 2794 (3) Å^3^	0.19 × 0.18 × 0.10 mm
*Z* = 4	

### Data collection

**Table:**

Bruker SMART APEXII diffractometer	2742 independent reflections
Radiation source: fine-focus sealed tube	1666 reflections with *I* > 2σ
graphite	*R*_int_ = 0.119
ω scans	θ_max_ = 26.0°, θ_min_ = 2.4°
Absorption correction: multi-scan (*SADABS*; Sheldrick, 1996)	*h* = −21→21
*T*_min_ = 0.862, *T*_max_ = 0.924	*k* = −12→12
15579 measured reflections	*l* = −22→22

### Refinement

**Table:**

Refinement on *F*^2^	Primary atom site location: structure-invariant direct methods
Least-squares matrix: full	Secondary atom site location: difference Fourier map
*R*[*F*^2^ > 2σ(*F*^2^)] = 0.040	Hydrogen site location: inferred from neighbouring sites
*wR*(*F*^2^) = 0.100	H-atom parameters constrained
*S* = 0.97	*w* = 1/[σ^2^(*F*_o_^2^) + (0.0417*P*)^2^] where *P* = (*F*_o_^2^ + 2*F*_c_^2^)/3
2742 reflections	(Δ/σ)_max_ < 0.001
186 parameters	Δρ_max_ = 0.60 e Å^−^^3^
0 restraints	Δρ_min_ = −0.55 e Å^−^^3^

### Special details

**Table:**

Geometry. All e.s.d.'s (except the e.s.d. in the dihedral angle between two l.s. planes) are estimated using the full covariance matrix. The cell e.s.d.'s are taken into account individually in the estimation of e.s.d.'s in distances, angles and torsion angles; correlations between e.s.d.'s in cell parameters are only used when they are defined by crystal symmetry. An approximate (isotropic) treatment of cell e.s.d.'s is used for estimating e.s.d.'s involving l.s. planes.
Refinement. Refinement of *F*^2^ against ALL reflections. The weighted *R*-factor *wR* and goodness of fit *S* are based on *F*^2^, conventional *R*-factors *R* are based on *F*, with *F* set to zero for negative *F*^2^. The threshold expression of *F*^2^ > σ(*F*^2^) is used only for calculating *R*-factors(gt) *etc*. and is not relevant to the choice of reflections for refinement. *R*-factors based on *F*^2^ are statistically about twice as large as those based on *F*, and *R*- factors based on ALL data will be even larger.

### Fractional atomic coordinates and isotropic or equivalent isotropic displacement parameters (Å^2^)

**Table:**

	*x*	*y*	*z*	*U*_iso_*/*U*_eq_	
Co1	0.0000	0.87355 (6)	0.2500	0.0423 (2)	
S1	−0.23413 (6)	0.57257 (9)	0.08021 (6)	0.0655 (3)	
O1	0.08519 (16)	0.8442 (2)	0.02024 (13)	0.0694 (7)	
N1	−0.00183 (16)	0.8085 (3)	0.08164 (14)	0.0517 (7)	
H1A	−0.0481	0.7640	0.0762	0.062*	
N2	0.03351 (16)	0.8912 (2)	0.14860 (13)	0.0429 (6)	
N3	0.09438 (15)	1.0297 (2)	0.28764 (13)	0.0416 (6)	
N4	−0.09755 (18)	0.7417 (3)	0.18238 (16)	0.0555 (7)	
C1	−0.1101 (2)	0.6416 (3)	−0.05536 (17)	0.0510 (8)	
H1	−0.1368	0.6775	−0.0239	0.061*	
C2	−0.1537 (2)	0.5487 (4)	−0.1162 (2)	0.0620 (10)	
H2	−0.2110	0.5218	−0.1269	0.074*	
C3	−0.1155 (3)	0.4944 (4)	−0.1620 (2)	0.0651 (10)	
H3	−0.1463	0.4302	−0.2034	0.078*	
C4	−0.0334 (2)	0.5333 (4)	−0.1474 (2)	0.0610 (10)	
H4	−0.0070	0.4963	−0.1788	0.073*	
C5	0.0115 (2)	0.6270 (3)	−0.08666 (18)	0.0532 (9)	
H5	0.0687	0.6536	−0.0768	0.064*	
C6	−0.0261 (2)	0.6823 (3)	−0.04033 (16)	0.0419 (7)	
C7	0.0246 (2)	0.7850 (3)	0.02159 (17)	0.0451 (8)	
C8	0.0888 (2)	0.9814 (3)	0.15504 (16)	0.0428 (7)	
H8	0.1071	0.9942	0.1130	0.051*	
C9	0.12177 (18)	1.0627 (3)	0.23016 (16)	0.0391 (7)	
C10	0.1779 (2)	1.1670 (3)	0.24127 (18)	0.0495 (8)	
H10	0.1959	1.1867	0.1998	0.059*	
C11	0.2070 (2)	1.2413 (3)	0.3128 (2)	0.0588 (9)	
H11	0.2442	1.3145	0.3210	0.071*	
C12	0.1812 (2)	1.2073 (4)	0.3725 (2)	0.0627 (10)	
H12	0.2013	1.2560	0.4230	0.075*	
C13	0.1256 (2)	1.1012 (3)	0.35803 (18)	0.0527 (9)	
H13	0.1089	1.0783	0.4000	0.063*	
C14	−0.1556 (2)	0.6713 (3)	0.13930 (19)	0.0472 (8)	

### Atomic displacement parameters (Å^2^)

**Table:**

	*U*^11^	*U*^22^	*U*^33^	*U*^12^	*U*^13^	*U*^23^
Co1	0.0530 (4)	0.0434 (4)	0.0368 (3)	0.000	0.0260 (3)	0.000
S1	0.0679 (7)	0.0601 (6)	0.0712 (6)	−0.0156 (5)	0.0344 (5)	−0.0118 (5)
O1	0.0732 (17)	0.093 (2)	0.0596 (13)	−0.0341 (16)	0.0455 (13)	−0.0249 (14)
N1	0.0644 (18)	0.0570 (18)	0.0456 (13)	−0.0208 (15)	0.0355 (13)	−0.0168 (14)
N2	0.0544 (17)	0.0446 (16)	0.0325 (12)	−0.0039 (14)	0.0225 (12)	−0.0067 (13)
N3	0.0454 (15)	0.0473 (16)	0.0335 (12)	0.0040 (13)	0.0196 (11)	−0.0042 (12)
N4	0.0649 (19)	0.0545 (19)	0.0543 (15)	−0.0096 (17)	0.0336 (15)	−0.0041 (16)
C1	0.055 (2)	0.059 (2)	0.0415 (15)	−0.0055 (19)	0.0240 (15)	−0.0068 (17)
C2	0.057 (2)	0.072 (3)	0.0566 (19)	−0.017 (2)	0.0256 (18)	−0.015 (2)
C3	0.075 (3)	0.063 (3)	0.0542 (19)	−0.008 (2)	0.028 (2)	−0.017 (2)
C4	0.075 (3)	0.063 (2)	0.0526 (19)	0.000 (2)	0.0364 (19)	−0.013 (2)
C5	0.057 (2)	0.064 (2)	0.0481 (17)	−0.0047 (19)	0.0329 (17)	−0.0077 (19)
C6	0.0475 (19)	0.0446 (19)	0.0324 (14)	−0.0012 (17)	0.0173 (14)	−0.0009 (15)
C7	0.053 (2)	0.049 (2)	0.0381 (15)	0.0017 (18)	0.0249 (15)	0.0019 (16)
C8	0.0485 (19)	0.0461 (19)	0.0376 (15)	−0.0008 (17)	0.0231 (14)	−0.0006 (15)
C9	0.0389 (18)	0.0405 (19)	0.0362 (14)	0.0056 (16)	0.0160 (14)	0.0004 (14)
C10	0.053 (2)	0.046 (2)	0.0452 (16)	−0.0013 (18)	0.0194 (16)	0.0016 (17)
C11	0.055 (2)	0.051 (2)	0.0608 (19)	−0.0083 (19)	0.0193 (17)	−0.008 (2)
C12	0.061 (2)	0.063 (3)	0.0556 (19)	−0.006 (2)	0.0192 (18)	−0.027 (2)
C13	0.055 (2)	0.059 (2)	0.0439 (16)	0.0025 (19)	0.0236 (16)	−0.0093 (18)
C14	0.056 (2)	0.044 (2)	0.0516 (18)	−0.0009 (18)	0.0335 (17)	0.0049 (17)

### Geometric parameters (Å, °)

**Table:**

Co1—N4^i^	2.054 (3)	C2—H2	0.9500
Co1—N4	2.054 (3)	C3—C4	1.364 (4)
Co1—N3	2.150 (3)	C3—H3	0.9500
Co1—N3^i^	2.150 (3)	C4—C5	1.389 (4)
Co1—N2	2.153 (3)	C4—H4	0.9500
Co1—N2^i^	2.153 (3)	C5—C6	1.386 (4)
S1—C14	1.624 (4)	C5—H5	0.9500
O1—C7	1.211 (3)	C6—C7	1.486 (4)
N1—N2	1.363 (3)	C8—C9	1.459 (4)
N1—C7	1.369 (3)	C8—H8	0.9500
N1—H1A	0.8801	C9—C10	1.389 (4)
N2—C8	1.289 (4)	C10—C11	1.373 (4)
N3—C13	1.340 (3)	C10—H10	0.9500
N3—C9	1.358 (3)	C11—C12	1.379 (4)
N4—C14	1.182 (4)	C11—H11	0.9500
C1—C2	1.380 (4)	C12—C13	1.389 (4)
C1—C6	1.397 (4)	C12—H12	0.9500
C1—H1	0.9500	C13—H13	0.9500
C2—C3	1.381 (4)		
			
N4^i^—Co1—N4	97.62 (16)	C2—C3—H3	120.1
N4^i^—Co1—N3	90.99 (11)	C3—C4—C5	120.1 (3)
N4—Co1—N3	164.56 (8)	C3—C4—H4	120.0
N4^i^—Co1—N3^i^	164.56 (8)	C5—C4—H4	120.0
N4—Co1—N3^i^	90.99 (11)	C4—C5—C6	120.7 (3)
N3—Co1—N3^i^	83.68 (13)	C4—C5—H5	119.6
N4^i^—Co1—N2	95.39 (10)	C6—C5—H5	119.6
N4—Co1—N2	90.98 (10)	C5—C6—C1	118.9 (3)
N3—Co1—N2	75.41 (9)	C5—C6—C7	117.7 (3)
N3^i^—Co1—N2	97.25 (9)	C1—C6—C7	123.3 (3)
N4^i^—Co1—N2^i^	90.98 (10)	O1—C7—N1	121.6 (3)
N4—Co1—N2^i^	95.39 (10)	O1—C7—C6	123.4 (2)
N3—Co1—N2^i^	97.25 (9)	N1—C7—C6	115.0 (3)
N3^i^—Co1—N2^i^	75.41 (9)	N2—C8—C9	116.6 (2)
N2—Co1—N2^i^	170.33 (14)	N2—C8—H8	121.7
N2—N1—C7	129.1 (2)	C9—C8—H8	121.7
N2—N1—H1A	115.5	N3—C9—C10	122.7 (3)
C7—N1—H1A	115.4	N3—C9—C8	116.0 (3)
C8—N2—N1	122.3 (2)	C10—C9—C8	121.3 (3)
C8—N2—Co1	117.08 (18)	C11—C10—C9	119.4 (3)
N1—N2—Co1	120.63 (18)	C11—C10—H10	120.3
C13—N3—C9	116.9 (3)	C9—C10—H10	120.3
C13—N3—Co1	128.18 (19)	C10—C11—C12	118.5 (3)
C9—N3—Co1	114.80 (18)	C10—C11—H11	120.7
C14—N4—Co1	175.8 (2)	C12—C11—H11	120.7
C2—C1—C6	119.4 (3)	C11—C12—C13	119.4 (3)
C2—C1—H1	120.3	C11—C12—H12	120.3
C6—C1—H1	120.3	C13—C12—H12	120.3
C1—C2—C3	121.2 (3)	N3—C13—C12	123.0 (3)
C1—C2—H2	119.4	N3—C13—H13	118.5
C3—C2—H2	119.4	C12—C13—H13	118.5
C4—C3—C2	119.7 (3)	N4—C14—S1	179.0 (3)
C4—C3—H3	120.1		
			
C7—N1—N2—C8	14.7 (5)	C4—C5—C6—C7	−178.0 (3)
C7—N1—N2—Co1	−164.3 (2)	C2—C1—C6—C5	−0.6 (4)
N4^i^—Co1—N2—C8	−90.9 (2)	C2—C1—C6—C7	177.7 (3)
N4—Co1—N2—C8	171.3 (2)	N2—N1—C7—O1	−3.1 (5)
N3—Co1—N2—C8	−1.3 (2)	N2—N1—C7—C6	176.6 (3)
N3^i^—Co1—N2—C8	80.2 (2)	C5—C6—C7—O1	18.1 (4)
N4^i^—Co1—N2—N1	88.1 (2)	C1—C6—C7—O1	−160.3 (3)
N4—Co1—N2—N1	−9.6 (2)	C5—C6—C7—N1	−161.6 (3)
N3—Co1—N2—N1	177.7 (2)	C1—C6—C7—N1	20.1 (4)
N3^i^—Co1—N2—N1	−100.8 (2)	N1—N2—C8—C9	−179.7 (2)
N4^i^—Co1—N3—C13	−85.0 (3)	Co1—N2—C8—C9	−0.7 (3)
N4—Co1—N3—C13	150.9 (3)	C13—N3—C9—C10	−1.4 (4)
N3^i^—Co1—N3—C13	80.5 (2)	Co1—N3—C9—C10	175.5 (2)
N2—Co1—N3—C13	179.7 (3)	C13—N3—C9—C8	178.4 (3)
N2^i^—Co1—N3—C13	6.1 (3)	Co1—N3—C9—C8	−4.6 (3)
N4^i^—Co1—N3—C9	98.5 (2)	N2—C8—C9—N3	3.6 (4)
N4—Co1—N3—C9	−25.7 (5)	N2—C8—C9—C10	−176.5 (3)
N3^i^—Co1—N3—C9	−96.0 (2)	N3—C9—C10—C11	−0.4 (5)
N2—Co1—N3—C9	3.18 (19)	C8—C9—C10—C11	179.7 (3)
N2^i^—Co1—N3—C9	−170.40 (19)	C9—C10—C11—C12	1.7 (5)
C6—C1—C2—C3	0.7 (5)	C10—C11—C12—C13	−1.2 (5)
C1—C2—C3—C4	−0.4 (5)	C9—N3—C13—C12	2.0 (4)
C2—C3—C4—C5	0.1 (5)	Co1—N3—C13—C12	−174.5 (2)
C3—C4—C5—C6	−0.1 (5)	C11—C12—C13—N3	−0.8 (5)
C4—C5—C6—C1	0.3 (5)		

Symmetry codes: (i) −*x*, *y*, −*z*+1/2.

### Hydrogen-bond geometry (Å, °)

**Table:**

*D*—H···*A*	*D*—H	H···*A*	*D*···*A*	*D*—H···*A*
C13—H13···O1^ii^	0.95	2.50	3.335 (5)	146

Symmetry codes: (ii) *x*, −*y*+2, *z*+1/2.
